# Effect of High Temperature Precipitation and Heating Dissolution on Microstructure and Mechanical Properties of Alloy 2618

**DOI:** 10.3390/ma19050903

**Published:** 2026-02-27

**Authors:** Yuan Yao, Jianhua Wang, Xuping Su, Ya Liu, Cengjie Shi, Shiyun He, Zhiwei Li

**Affiliations:** 1 Jiangsu Key Laboratory of Materials Surface Science and Technology, Changzhou University, Changzhou 213164, China; 15161106236@163.com (Y.Y.); sxping@cczu.edu.cn (X.S.); yliu@cczu.edu.cn (Y.L.); lzw@cczu.edu.cn (Z.L.); 2 School of Petroleum and Natural Gas Engineering, Changzhou University, Changzhou 213164, China; 15189281684@163.com (C.S.); 17883634063@163.com (S.H.)

**Keywords:** alloy 2618, high-temperature precipitation, heating dissolution, precipitation-free zone, mechanical properties

## Abstract

This study employs high-temperature precipitation combined with heating dissolution to redistribute solute atoms near the grain boundaries of alloy 2618, regulating the width of the precipitation-free zone at the grain boundaries and the aging precipitates in their vicinity. Microscopy techniques, including high-resolution scanning electron microscopy and transmission electron microscopy, were used to observe the grain-boundary structure of the alloy. A universal electronic tensile testing machine and an impact tester were used to evaluate the mechanical properties of the alloy. The results show that solution treatment at 535 °C for 30 min, followed by high-temperature precipitation at 470 °C for 10 min and subsequent heating dissolution at 535 °C for 10 min, significantly narrowed the width of the precipitation-free zone at the grain boundaries of alloy 2618. The number of precipitated phases in the vicinity of the grain boundaries increased. Compared with the conventional solution aging treatment of alloy 2618, the tensile strength and impact toughness of the alloy subjected to high-temperature precipitation, heating dissolution, and aging increased by 5.0% and 23.7%, respectively. Thus, the synergistic effects of high-temperature precipitation and heating dissolution effectively improved the grain-boundary structure and enhanced the overall mechanical properties of alloy 2618.

## 1. Introduction

Alloy 2618, composed of Al, Cu, Mg, Fe, and Ni, is strengthened by the Al_9_FeNi phase and the S′ (Al_2_CuMg) precipitate phase [1]. It is widely used in critical load-bearing components that require high strength and operate at temperatures above 150 °C, such as the cockpit central floor of supersonic fighter jets and automotive engine blades [2]. The width of the precipitation-free zone (PFZ) at the grain boundaries and the distribution of precipitated phases within the grain interior significantly influence the mechanical properties of alloy 2618. The S′ precipitates within the grain interior undergo significant growth and coarsening at high temperatures, gradually transforming into the S phase, which markedly reduces their strengthening effect [3]. The formation of precipitation-free zones at the grain boundaries can be attributed to the vacancy depletion theory and the solute depletion theory [4]. The vacancy depletion theory [5] proposes that grain boundaries, as surface defects, can absorb dislocations and vacancies. This reduces the vacancy concentration near the grain boundary below the critical level required for the nucleation of precipitates, leading to the formation of PFZs. The solute depletion theory [6] suggests that the nucleation and growth of precipitates at the grain boundaries consume solute elements in adjacent regions. The resulting reduction in solute supersaturation leads to the formation of PFZs. The formation of PFZs is closely related to the heat treatment process of the alloys. Chen [7] showed that the evolution of PFZs during alloy homogenization can be divided into three stages: PFZ formation and decay, PFZ dissolution, and PFZ reproduction and broadening. Okuda [8] employed Monte Carlo simulations to investigate the effect of grain boundary precipitation on the microstructure near the PFZ during aging treatment. When solute depletion at the grain boundary and precipitation within the grain interior occur simultaneously, the average size of precipitated phases near the PFZ is nearly constant. Ren [9] showed that higher aging temperatures led to wider PFZ in the alloy, whereas at a constant aging temperature, the width of the PFZ does not increase with prolonged aging time.

High-temperature precipitation is a heat treatment process in which an aluminum alloy is held at a temperature slightly below the solvus of second-phase particles for a certain period after solution treatment, leading to the precipitation of equilibrium phases. Chen [10] found that high-temperature precipitation can effectively improve the stress corrosion resistance and mechanical properties of 7A52-T4 and 7A52-T6 alloys. Xinming Zhang [11] found that high-temperature precipitation can affect the size and distribution of grain boundary precipitates in 7A55 aluminum alloy. The discontinuous distribution of the phase η effectively blocks corrosion channels, thereby improving the corrosion resistance of the alloy. Li [12] reported the high-temperature precipitation treatment of 7050 aluminum alloy at 355 °C for 0.5 h, which resulted in the formation of numerous fine precipitates within the grain.

In sustained high temperature environments, 2618 alloy exhibits good structural stability and resistance to degradation. The Ni and Fe elements in the alloy form dispersed Al9FeNi and other thermally stable strengthening phases, effectively suppressing grain growth and coarsening of precipitates, and slowing down the high-temperature softening process. After exposure to high temperature of 250 °C for 100 h, it can still maintain a high instantaneous tensile strength. Especially for modified alloys containing rare earth elements such as Zr, the performance degradation is slower [13]. 2618 alloy has strong creep resistance under constant high temperature and load. In the temperature range of 160–200 °C, its creep stress index is high, the deformation rate is slow, the fracture time is long, and it is suitable for bearing long-term static or dynamic loads [14]. Under frequent start stop thermal cycling conditions in aircraft engine components, the material exhibits low stress relaxation and stable cyclic softening behavior, with controllable crack initiation and propagation rates. Endurance tests conducted under specific temperature and stress conditions have shown that 2618 alloy can maintain a long period of time without fracture at high temperatures, providing support for predicting service life. The current stage of research has proven that the optimized 2618 alloy can maintain good performance at high temperatures, but further experimental research and engineering application verification are still essential [15].

The precipitates at the grain boundaries or sub-grain boundaries are relatively large and distributed in a net-like pattern. Automotive turbocharged engine blades typically operate for extended periods at temperatures above 200 °C, which places high demands on the high-temperature mechanical properties of 2618 aluminum alloy. Based on the solute depletion theory of PFZ formation, this study applies high-temperature precipitation and heating dissolution treatments to alloy 2618. By controlling the solute concentration in the grain boundary region, the grain boundary microstructure and mechanical properties of the alloy can be improved [16]. The results of this study will provide an important reference for improving the mechanical properties of alloy 2618 and expanding its application range.

## 2. Materials and Methods

The raw materials used in this experiment were extruded alloy 2618 rods (Φ10 mm × 100 mm) supplied by Shanghai Weilande Aluminum Co., Ltd. (Shanghai, China). The chemical composition of the alloy is detailed in Table 1.

High-temperature precipitation: Alloy 2618 rods (Φ10 mm × 100 mm) were first solution-treated at 535 °C for 30 min in a Sk2-4-10 tubular resistance furnace. Subsequently, the samples were cooled at a rate of 2 °C/min to 500, 485, 470, 455, and 440 °C, respectively, and held for 0–20 min to perform high-temperature precipitation. The samples were then immediately quenched in water. Finally, the optimal high-temperature precipitation parameters were determined through microstructural observation and impact toughness testing.

Heating dissolution: Alloy 2618 rods (Φ10 mm × 100 mm) were first solution-treated at 535 °C for 30 min in a Sk2-4-10 tubular resistance furnace. Subsequently, the samples were cooled at a rate of 2 °C/min to the optimal high-temperature precipitation temperature. The temperature was then increased to 535 °C at a rate of 4 °C/min and held for 0, 5, 10, and 20 min, respectively, to perform heating dissolution. Finally, the samples were immediately quenched in water. The optimal heating dissolution time was determined through microstructural analysis and impact toughness testing.

Aging treatment: After high-temperature precipitation and heating dissolution, the alloy 2618 rods were aged at 200 °C for different durations. The peak aging time was determined based on the Vickers hardness test.

To prevent oxidation of the sample at high temperatures, high-purity argon (Ar, 99.99%) is used as the protective atmosphere. Argon, an inert gas, does not participate in reactions. With a density greater than air, it naturally descends and displaces the existing air when introduced into the furnace, creating a localized inert environment around the sample to achieve physical isolation from oxygen.

Operation Procedure Description: Before heating, introduce argon gas into the furnace at a flow rate of 500 mL/min for 15 min to fully replace the internal air. During heating and holding, reduce the flow rate to and maintain it at 200 mL/min to ensure a slight positive pressure inside the furnace, preventing external air from being drawn back. During the aging phase, continue argon flow until the process is completed to avoid oxidation of the sample in the low-temperature reflux air.

Metallographic samples were fabricated from alloy 2618 test rods following high-temperature precipitation, heating dissolution, and subsequent aging. The samples were subjected to rough grinding, fine grinding, and polishing treatments in sequence and then etched using Keller’s reagent. The microstructure of alloy 2618 was observed using a DMI3000M optical microscope (Shanghai, China). A JEOL-6510 scanning electron microscope (Beijing, China) equipped with an energy-dispersive spectrometer was used to analyze the morphology, distribution, and elemental composition of the phases in alloy 2618. The MTP-1A dual-spray electrolytic thinning instrument (Guangzhou China) was used to thin the circular sample to a thickness of 80 μm and a diameter of 3 mm. The dual-spray electrolytic solution consisted of 30% HNO_3_ and 70% CH_3_OH. The current and voltage were set to 50–70 mA and 10–20 V, respectively. TECNAIG20 transmission electron microscope (Guangzhou China) was used to observe the precipitation phases within the grain and the grain-boundary microstructure of alloy 2618. Select 10 different grain boundaries for observation and measure their PFZ lengths separately and take the average value. Select representative grain boundaries for line scanning and observe changes in element content near the grain boundaries; And perform point scanning on the precipitates near the grain boundaries to determine their composition.

The impact test specimens were prepared according to ISO 148-1, and the impact toughness of alloy 2618 was measured with a TSKL-300J pendulum impact testing machine (Shanghai, China). The Vickers hardness of the samples was measured with an HXD-1000TMC/LC hardness tester. A load of 50 N was applied for 15 s during the Vickers hardness test. The reported hardness represents the average of six measurements taken at different locations. Tensile test specimens were prepared according to ISO 6892-1, with a 10 mm diameter and a 30 mm gauge length. Tensile tests were conducted using a WDT-030 microcomputer-controlled universal testing machine at a strain rate of 2 mm/min. The reported tensile values represent the average of three measurements.

## 3. Results and Discussion

### 3.1. Optimization of High-Temperature Precipitation Parameters

**Figure 1:**
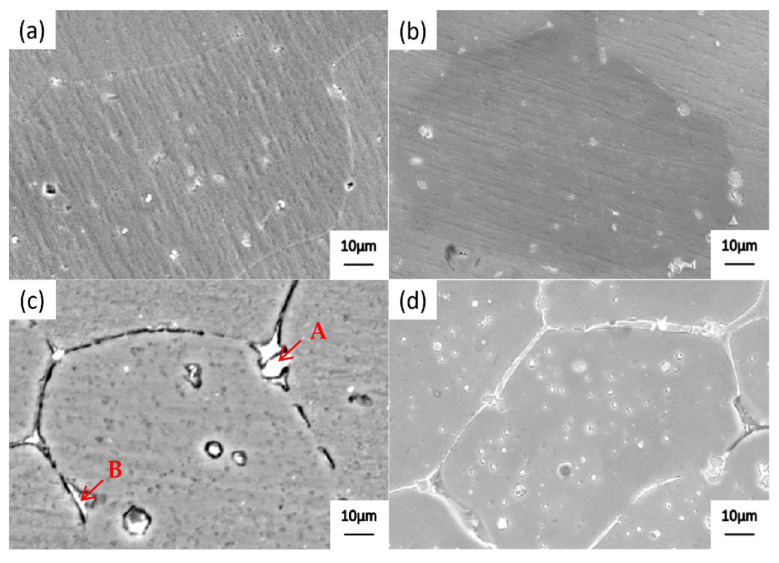
SEM images of alloy 2618 after precipitation at different temperatures for 10 min. (**a**) 500 °C; (**b**) 485 °C; (**c**) 470 °C, and Point A and B are used for energy spectrum analysis; (**d**) 455 °C.

Figure 1 shows the scanning electron microscopy (SEM) microstructure of alloy 2618 after solution treatment at 535 °C for 30 min and high-temperature precipitation for 10 min. At a precipitation temperature of 500 °C, a small amount of precipitate forms within the grain and at the grain boundaries of alloy 2618 (Figure 1a). When the precipitation temperature is lowered to 485 °C, the quantity and particle size of precipitated phases at the grain boundaries increase (Figure 1b). When the precipitation temperature is lowered to 470 °C, the precipitates with the grain and at the grain boundaries undergo significant growth (Figure 1c). At a precipitation temperature of 455 °C, a large number of precipitated phases form within the grain and at the grain boundaries (Figure 1d). These precipitated phases are continuously distributed at grain boundaries.

**Figure 2:**
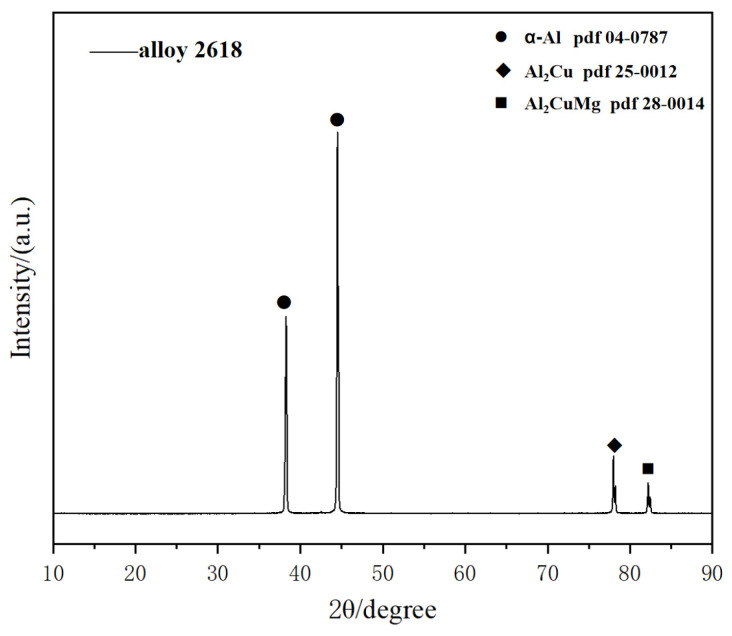
XRD images of alloy 2618 after precipitation at 470 °C for 10 min.

The energy spectrum analysis of the precipitated phase at the grain boundary in Figure 1c is summarized in Table 2, revealing the presence of Al_2_Cu and Al_2_CuMg phases. XRD images of alloy 2618 after precipitation at 470 °C for 10 min can also prove it (Figure 2).

**Figure 3:**
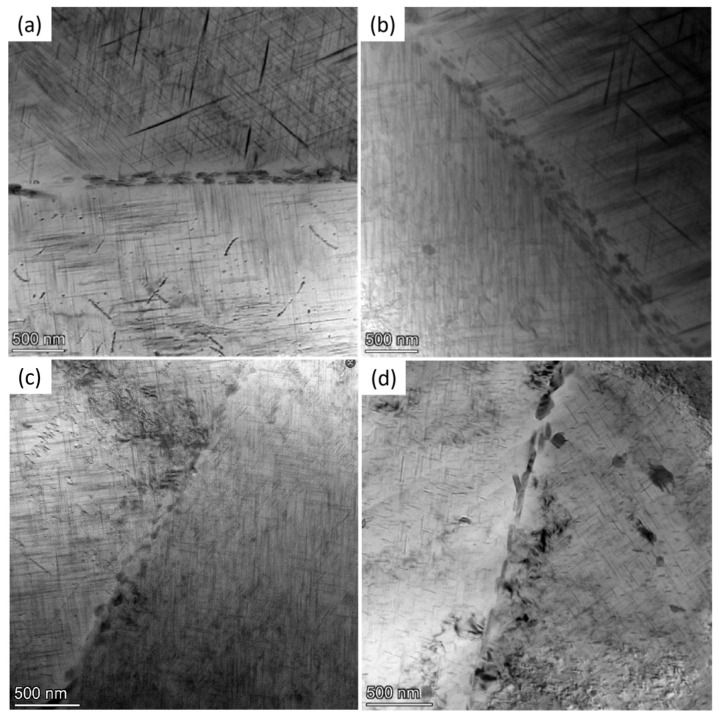
TEM images of alloy 2618 after precipitation at different temperatures for 10 min: (**a**) 500 °C; (**b**) 485 °C; (**c**) 470 °C; (**d**) 455 °C.

Figure 3 shows the transmission electron microscopy (TEM) microstructure of alloy 2618 after solution treatment at 535 °C for 30 min and high-temperature precipitation for 10 min. This allows for clearer observation of the distribution, size, and morphology of precipitates within the grain and at the grain boundaries.

When the precipitation temperature is 500 °C, numerous fine needle-shaped precipitates are present within the grains of alloy 2618, with granular precipitates appearing at the grain boundaries (Figure 3a). These precipitated phases occupy a relatively narrow region. When the precipitation temperature is lowered to 485 °C, the needle-shaped precipitates within the grain become coarser (Figure 3b). The number and particle size of granular precipitates at the grain boundaries increase significantly. In addition, the width of the grain boundary region occupied by these precipitated phases increases markedly. As shown in Figure 3c, when the precipitation temperature is lowered to 470 °C, the long needle-shaped precipitates within the grain transform into short needle-shaped or granular phases. The precipitated phases at the grain boundaries undergo coarsening but remain intermittently distributed along the grain boundaries. The width of the grain boundary region occupied by these precipitated phases begins to decrease. When the precipitation temperature is further lowered to 455 °C, most of the long needle-shaped precipitates within the grain transform into short needle-shaped or granular phases, while particularly coarse granular precipitates simultaneously appear (Figure 3d). In addition, the precipitated phases at the grain boundaries undergo significant coarsening, and the precipitates become nearly end-to-end connected, resulting in a continuous distribution at the grain boundaries. Due to the formation of coarse precipitates, solute atoms in the surrounding region are depleted and PFZ appear near the grain boundary.

High-temperature precipitation utilizes the tendency of alloy grain boundaries to preferentially precipitate phases, allowing solute atoms within the grains to diffuse towards the grain boundaries. During the heating process, the precipitated phase undergoes re-dissolution, increasing the solute concentration at and near the grain boundaries and reducing the width of the PFZ. The quantity, size, and morphology of grain boundary precipitates have a significant influence on subsequent dissolution during heating. Only a limited number of precipitated phases is required at the grain boundaries to ensure their rapid dissolution into the matrix during heating and holding. Therefore, considering the control of grain boundary precipitate quantity, size, and morphology, the optimal precipitation temperature for alloy 2618 was identified as 470 °C.

Grain boundaries are regions where various defects are concentrated and exhibit many characteristics distinct from those within the grains. Atoms at the grain boundaries deviate from their equilibrium positions and possess high potential energy. By slightly adjusting the position of atoms on the grain boundary, a new phase interface can be formed. The Gibbs free energy barrier for the nucleation of precipitated phases at the grain boundaries is lower than that within the grain. In addition, solute atoms diffuse more rapidly along grain boundaries, facilitating nucleation and the growth of new phases at the grain boundaries compared to within the grain. During the high-temperature precipitation of alloy 2618, the driving force for the formation of new phases at the grain boundaries is small, which limits precipitation within the grain. This creates favorable conditions for the preferential precipitation of new phases at the grain boundaries. In addition, solute atoms can continuously diffuse to the grain boundary precipitates at high temperatures, resulting in the formation of coarse granular precipitates at the grain boundaries.

When alloy 2618 undergoes high-temperature precipitation, the nucleation and growth of new phases are not significantly constrained by atomic diffusion kinetics. The thermodynamic driving force and solute supersaturation in the matrix are the main factors influencing the nucleation and growth of new phases. As the precipitation temperature decreases, the driving force for the formation of precipitates and the degree of supersaturation in the matrix increase, promoting the continuous growth and coarsening of precipitates.

**Figure 4:**
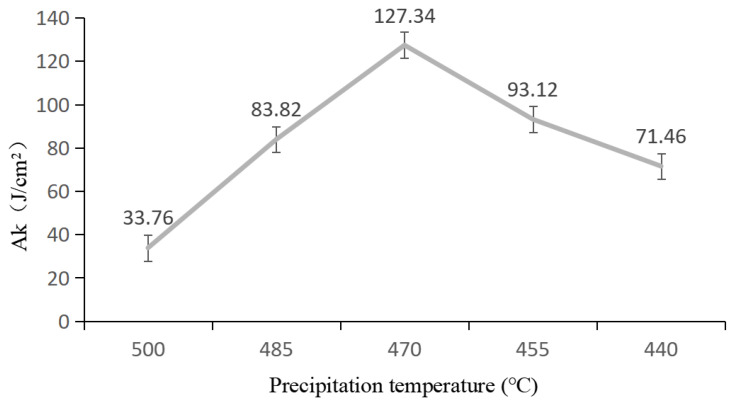
Relationship between the impact toughness of alloy 2618 and its precipitation temperature.

Figure 4 shows the relationship between the impact toughness of alloy 2618 and its precipitation temperature. As the precipitation temperature decreases, the impact toughness of alloy 2618 initially increases rapidly, reaching a maximum at 470 °C, and then gradually decreases (Figure 4). The impact energy of an alloy reflects a combination of its tensile strength and elongation at break. If the tensile strength and elongation at break are both high, the impact energy of the alloy will be relatively high [17]. Alloy 2618 is strengthened mainly through two mechanisms: solid solution strengthening and precipitation strengthening [18]. Since the strengthening of alloy 2618 primarily occurs through aging phases, the effect of precipitation strengthening is significantly greater than that of solid solution strengthening. When alloy 2618 is solution-treated at 535 °C for 30 min, copper and magnesium are largely dissolved in the matrix. At this stage, the primary strengthening mechanism is solid solution strengthening. When alloy 2618 is cooled to 500 °C for precipitation, the number of precipitates within the grain and at the grain boundaries remains relatively small, as the temperature is only slightly below the solid solution line. At this stage, the alloy is still mainly strengthened by solid solution, so the impact energy of alloy 2618 is relatively low. When alloy 2618 is cooled to 485 °C for precipitation, the supersaturation of solute atoms in the matrix increases and a greater number of precipitated phases appear within the grain and at the grain boundaries. Due to the increased strengthening effect of the precipitated phases, the impact energy of the alloy is significantly increased. When alloy 2618 is cooled to 470 °C for precipitation, the number of precipitated phases within the grain and at the grain boundaries further increases, leading to a corresponding rise in the impact energy of the alloy. At this stage, the impact toughness of the alloy reaches its maximum. As the precipitation temperature decreases to 455 and 440 °C, the number of precipitates within the grain and at grain boundaries increases; however, the coarsening of precipitates and the continuous distribution of grain boundary precipitates result in a continuous decrease in the impact toughness of the alloy. In addition, PFZs appeared near the grain boundaries. The presence of PFZs at the grain boundaries also contributed to the reduced impact toughness of the alloy [19]. Therefore, based on the impact toughness of alloy 2618, the optimal precipitation temperature is 470 °C.

**Figure 5:**
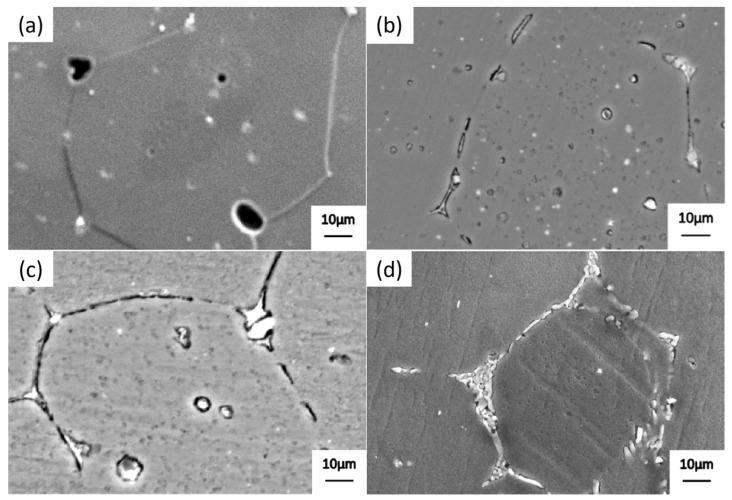
SEM images of alloy 2618 after precipitation at 470 °C for different durations: (**a**) 0 min; (**b**) 5 min; (**c**) 10 min; (**d**) 20 min.

Figure 5 shows the SEM microstructure of alloy 2618 after precipitation at 470 °C for different durations. When the temperature of alloy 2618 was decreased from 535 to 470 °C, almost no precipitated phases were observed at the grain boundaries (Figure 5a). When the alloy was held at 470 °C for 5 min, only a small amount of discontinuous, fine precipitates formed at the grain boundaries. Extending the precipitation time to 10 min led to an increase in the number and size of the precipitated phases at the grain boundaries. When the precipitation time was increased to 20 min, a large number of precipitation phases formed at the grain boundaries of the alloy. Furthermore, the precipitation phases were nearly continuously distributed along the grain boundaries. Therefore, to control the number of grain boundary precipitates and prevent their continuous distribution, the optimal precipitation time for alloy 2618 at 470 °C was set at 10 min.

### 3.2. Optimization of Heating Dissolution Time

**Figure 6:**
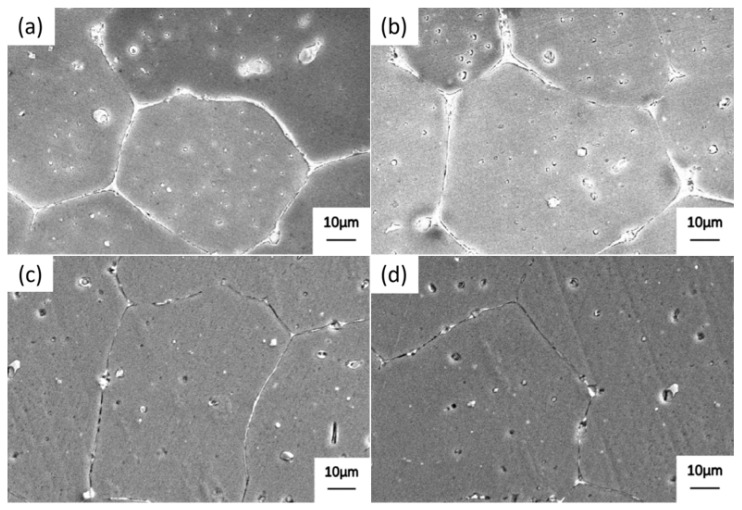
SEM images of alloy 2618 after high-temperature precipitation and heating dissolution at 535 °C for different durations: (**a**) 0 min; (**b**) 5 min; (**c**) 10 min; (**d**) 20 min.

Figure 6 shows the SEM microstructure of alloy 2618 after high-temperature precipitation and heating dissolution at 535 °C for different durations. As shown in Figure 6a, when the temperature is increased from 470 to 535 °C, supersaturated solute atoms diffuse from within the grain to the grain boundary at lower temperatures, resulting in the further formation of continuously distributed precipitated phases along the grain boundaries. As shown in Figure 6b, when alloy 2618 is held at 535 °C for 5 min, most of the precipitated phases at the interfaces between two adjacent grains undergo dissolution. However, coarse precipitates are still observed at the triple junction of these grains. As shown in Figure 6c, when the holding time is increased to 10 min, the majority of the precipitated phases at the grain boundaries undergo re-dissolution, leaving only a few undissolved precipitate particles. As shown in Figure 6d, when the holding time is increased to 20 min, the precipitated particles within the grain are slightly refined. Therefore, a heating dissolution time of 10 min is sufficient to effectively eliminate precipitate phases at the grain boundaries.

### 3.3. Microstructure and Mechanical Properties of Alloy 2618

**Figure 7:**
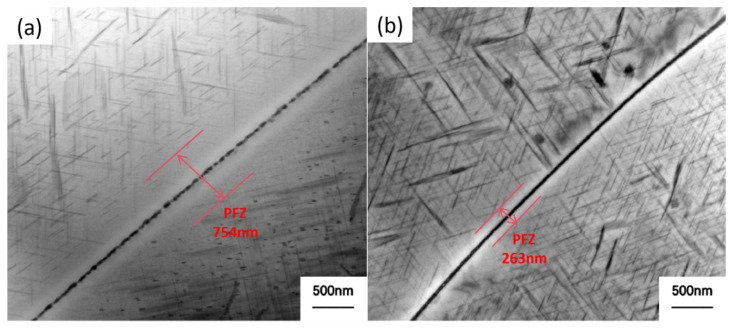
TEM images of peak-aged alloy 2618 after different solution treatments: (**a**) Conventional solid solution; (**b**) High-temperature precipitation followed by heating dissolution treatment.

Figure 7 shows the TEM images of peak-aged alloy 2618 after different solution treatments. Conventional solid followed by peak aging involves solution treatment at 535 °C for 30 min, water quenching, and aging at 200 °C for 11 h. High-temperature precipitation and heating dissolution solution treatment followed by peak aging includes solid solution treatment at 535 °C for 30 min, high-temperature precipitation at 470 °C for 10 min, heating dissolution at 535 °C for 10 min, water quenching, and aging at 200 °C for 11 h.

As shown in Figure 7a, the number of precipitated phases near the grain boundaries of alloy 2618 subjected to the conventional solid solution and peak aging process is relatively small, which are coarse and intermittently distributed. A wide PFZ of approximately 754 nm is observed near the grain boundary. As shown in Figure 7b, the precipitates near the grain boundaries of alloy 2618, subjected to high-temperature precipitation, solution treatment, and aging, are relatively dense. A thin layer of continuous precipitated phase forms at the grain boundary, and the width of the PFZ is significantly reduced to approximately 263 nm.

**Figure 8:**
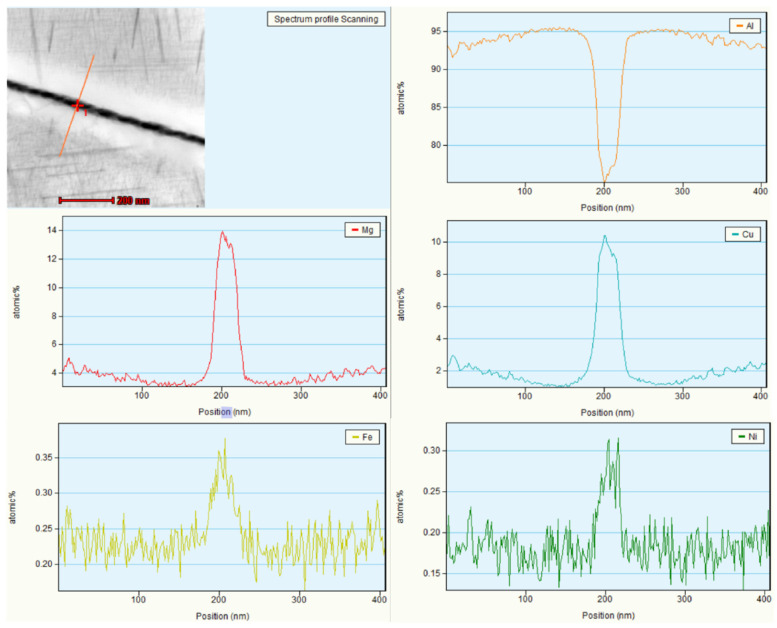
Line scan analysis near the grain boundary of alloy 2618.

To identify the precipitated phase at the grain boundary, energy dispersive analysis was conducted on the adjacent region. As shown in Figure 8, the precipitate regions along the grain boundaries exhibited decreased aluminum content, increased iron and nickel contents, and markedly increased copper and magnesium contents. The precipitates at the grain boundaries are likely primarily Al_2_Cu and Al_2_CuMg, with a small amount of Al_9_FeNi. In addition, compared with the matrix region far from the grain boundary, the region adjacent to the grain boundary precipitates lacks copper and magnesium, leading to the formation of PFZs. The formation and variation in width of PFZs at the grain boundaries of 2618 aluminum alloy can be explained by solute depletion theory.

**Figure 9:**
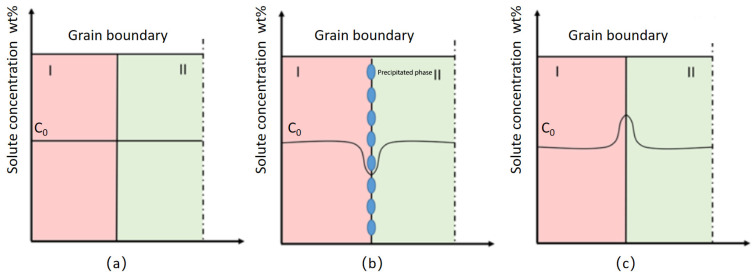
Distribution of solute concentration near the grain boundaries of alloy 2618 after different treatments: (**a**) Conventional solution treatment; (**b**) High-temperature precipitation; (**c**) Heating dissolution.

Figure 9 shows the solute concentration distribution near the grain boundary of alloy 2618 after solid solution treatment, high-temperature precipitation, and heating dissolution. As shown in Figure 9a, when alloy 2618 is subjected to solution treatment, the solute concentration within the grains and at the grain boundaries is essentially uniform. During the aging process, the S (Al_2_CuMg) phase preferentially nucleates and grows at the grain boundaries, significantly lowering the solute concentration in the surrounding regions. The reduction in solute supersaturation results in the formation of a relatively wide PFZ, as shown in Figure 7a. After high-temperature precipitation, the second phases in alloy 2618 preferentially nucleate and grow along the alloy grain boundaries (Figure 9b). At this stage, the solute concentration near the grain boundary will decrease. As shown in Figure 9c, during solution treatment, the second phases at the grain boundary will redissolve, allowing for the redistribution of solute atoms [20]. By controlling the re-dissolution time, the solute concentration near the grain boundary becomes higher than in regions farther away, creating a solute distribution that helps reduce the width of PFZs and aids in further improving the grain boundary structure of the alloy [21]. Therefore, the combination of high-temperature precipitation, solution treatment, and aging resulted in a significant reduction in the width of the PFZ in alloy 2618, as shown in Figure 7b.

**Figure 10:**
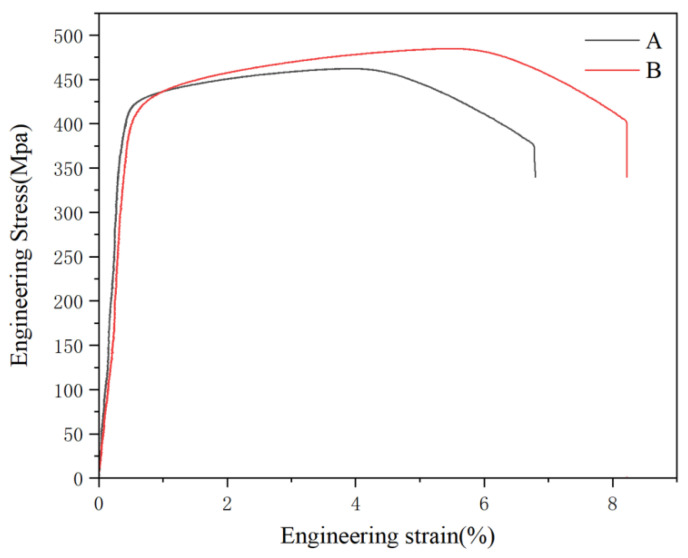
Stress–strain curve of peak-aged alloy 2618 after different solution treatments: (A) Conventional solid solution; (B) High-temperature precipitation followed by heating dissolution treatment.

As shown in Figure 10, curves A and B have similar slopes in the initial stage, indicating that the two samples have comparable elastic moduli in the elastic phase, meaning their resistance to elastic deformation is similar. Curve B exhibits a slightly higher yield stress than curve A, suggesting that sample B requires greater stress to initiate plastic deformation. The figure reveals that curve B shows a steeper stress increase during the strengthening phase compared to curve A, indicating more pronounced work hardening and greater strength improvement in sample B. Both curves A and B exhibit a necking stage, but the stress decline in curve B is relatively gentler, implying better toughness in sample B during necking, allowing it to withstand deformation without immediate fracture. Ultimately, the curves drop to zero as the material fractures. The figure demonstrates that curves A and B have different fracture strains, with curve B exhibiting a larger fracture strain, indicating better ductility in sample B and the ability to sustain greater deformation.

Table 3 presents the mechanical properties of peak-aged alloy 2618 after different solid solution treatments. As shown in Table 3, the tensile strength, elongation at break, and impact toughness of aged alloy 2618 treated with the conventional solution were 462 MPa, 10.6%, and 30.3 J/cm^2^, respectively. In comparison, these values increased by 5%, 1.9%, and 23.8%, respectively, following high-temperature precipitation and heating dissolution. These results indicate that the comprehensive mechanical properties of the alloy were significantly improved.

**Figure 11:**
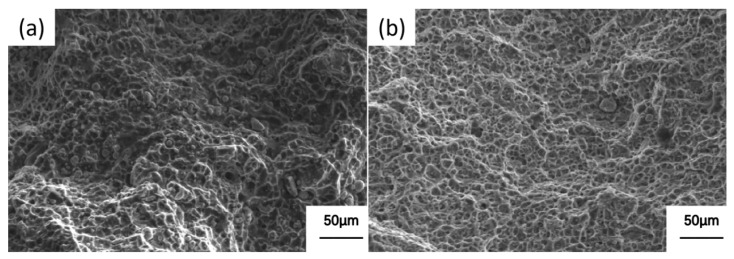
Tensile fracture morphologies of peak-aged alloy 2618 after different solid solution treatments: (**a**) Conventional solid solution; (**b**) High-temperature precipitation followed by heating dissolution treatment.

Figure 11 shows the tensile fracture morphologies of aged alloy 2618 after different solid solution treatments. As shown in Figure 11a, the tensile fracture surface of aged alloy 2618 subjected to conventional solid solution treatment exhibits numerous ductile dimples and tearing edges. In addition, many protruding particles are observed on the fracture surface, which adversely affect the toughness of the alloy [22]. In contrast, the tensile fracture surface of aged alloy 2618 after high-temperature precipitation and solution treatment showed numerous small dimples and fine tearing edges (Figure 11b). Compared with the tensile fracture of the aged alloy 2618 after conventional solution treatment, a greater number of smaller ductile dimples and tearing edges were observed. In addition, the number of protruding particles on the fracture surface was significantly reduced, as the extended solution treatment time allowed the second phases to dissolve sufficiently into the alloy matrix. The tensile fracture characteristics of aged alloy 2618 indicate that high-temperature precipitation and solution treatment improved its comprehensive mechanical properties [23].

## 4. Conclusions

(1) The optimal process parameters for the high-temperature precipitation and heating dissolution of alloy 2618 are as follows: solution treatment at 535 °C for 30 min, cooling to 470 °C and holding for 10 min for high-temperature precipitation, then heating to 535 °C and holding for 10 min for dissolution treatment.

(2) High-temperature precipitation followed by heating dissolution can significantly reduce the width of the precipitate-free zone at the grain boundaries of aged 2618 aluminum alloy while increasing the amount of precipitated phase in the regions near the grain boundaries.

(3) Compared with conventional solution-aging treatment, the high-temperature precipitation and heating dissolution aging of alloy 2618 increased the tensile strength and impact toughness by 5.0% and 23.7%, respectively.

## Figures and Tables

**Table 1:** Chemical composition of alloy 2618 (wt.%).

Cu	Mg	Fe	Ni	Si	Zn	Al
2.2	1.5	1.1	1.2	0.08	0.02	Bal.

**Table 2:** Energy spectrum analysis of precipitates at the grain boundaries of alloy 2618 (at.%).

Point	Cu	Mg	Fe	Ni	Al
A	25.91	2.74	0.1	0.1	71.14
B	23.33	2.62	0.08	0.08	73.89

**Table 3:** Mechanical properties of peak-aged alloy 2618 after different solid solution treatments.

Process	Impact Toughness (J/cm^2^)	Tensile Strength (MPa)	Elongation at Break (%)
Conventional solution treatment	30.3 ± 2.0	462 ± 5.0	10.6 ± 0.5
High-temperature precipitation and solution treatment	37.5 ± 2.0	485 ± 5.0	10.8 ± 0.5

## Data Availability

The original contributions presented in this study are included in the article. Further inquiries can be directed to the corresponding author.
